# Contrast-modulated stimuli produce more superimposition and predominate perception when competing with comparable luminance-modulated stimuli during interocular grouping

**DOI:** 10.1038/s41598-020-69527-5

**Published:** 2020-08-07

**Authors:** Jan Skerswetat, Monika A . Formankiewicz, Sarah J. Waugh

**Affiliations:** 1 Department of Vision and Hearing Sciences, Anglia Vision Research, Anglia Ruskin University, East Road, Cambridge, CB1 1PT UK; 2 Department of Psychology, Northeastern University, 360 Huntington Ave, Boston, MA 02115 USA

**Keywords:** Human behaviour, Pattern vision

## Abstract

Interocular grouping (IOG) is a binocular visual function that can arise during multi-stable perception. IOG perception was initiated using split-grating stimuli constructed from luminance (L), luminance-modulated noise (LM) and contrast-modulated noise (CM). In Experiment 1, three different visibility levels were used for L and LM (or first-order) stimuli, and compared to fixed-visibility CM (or second-order) stimuli. Eight binocularly normal participants indicated whether they perceived full horizontal or vertical gratings, superimposition, or other (piecemeal and eye-of-origin) percepts. CM stimuli rarely generated full IOG, but predominantly generated superimposition. In Experiment 2, Levelt’s modified laws were tested for IOG in nine participants. Split-gratings presented to each eye contained different visibility LM gratings, or LM and CM gratings. The results for the LM-vs-LM conditions mostly followed the predictions of Levelt’s modified laws, whereas the results for the LM-vs-CM conditions did not. Counterintuitively, when high-visibility LM and low-visibility CM split-gratings were used, high-visibility LM components did not predominate IOG perception. Our findings suggest that higher proportions of superimposition during CM-vs-CM viewing are due to binocular combination, rather than mutual inhibition. It implies that IOG percepts are more likely to be mediated at an earlier monocular, rather than a binocular stage. Our previously proposed conceptual framework for conventional binocular rivalry, which includes asymmetric feedback, visual saliency, or a combination of both (Skerswetat et al. Sci Rep 8:14432, 2018), might also account for IOG. We speculate that opponency neurons might mediate coherent percepts when dissimilar information separately enters the eyes.

## Introduction

The human visual system has evolved to combine monocular inputs into a coherent, fused, binocular percept if those inputs have similar physical properties. Significant physical differences between two monocular images can lead to exclusive visibility of one eye’s input for a few seconds until that eye’s input is suppressed, as the formerly suppressed eye’s input takes over visual awareness. This phenomenon is known as binocular rivalry^2–5^.

Conventional binocular rivalry (CBR) is typically generated by presenting for example, a vertical grating to one eye and a horizontal grating to the other. Perception will begin to alter between an exclusive vertical and an exclusive horizontal grating. Traditionally, CBR is known for alternation between two exclusive percepts but in transition between these exclusivity states, CBR also consists of “mixed” percepts, namely piecemeal (i.e. exclusivity in smaller local spatial zones across the visual field^6^) and superimposition (i.e. percept of completely overlapping rival stimuli^7^. Superimposition has been thought to be indicative of binocular fusion^7,8^.

**Figure 1:**
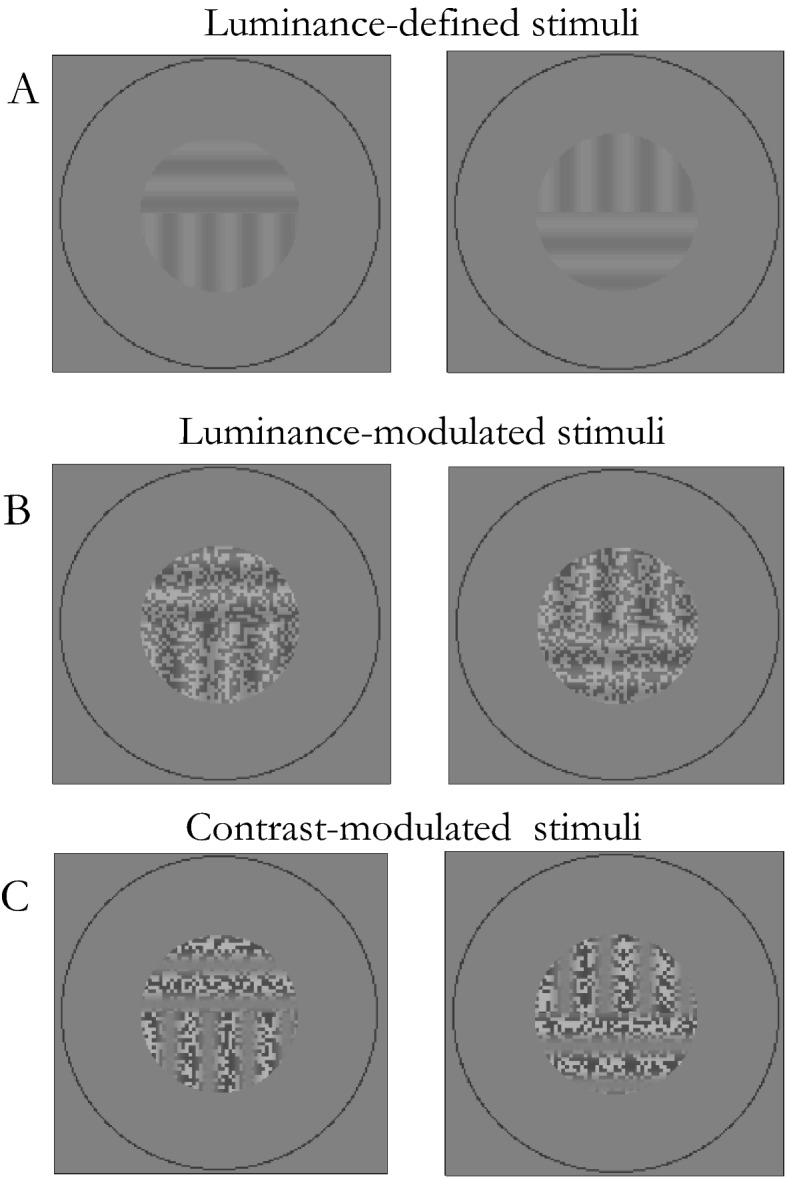
Example stimuli of comparable visibility used in the first experiment. (**A**) Luminance-defined (L) stimuli without noise. (**B**) Luminance-modulated (LM) noise stimuli. (**C**) Contrast-modulated (CM) noise stimuli. Orientations were counterbalanced across stimulus types and eyes. The reader may experience IOG by looking at the gratings held at a distance of approximately 30–40 cm. Position an index fingertip approximately halfway between the eyes and gratings, so that each eye views the index fingertip centrally for one of the two gratings or split-gratings. Now fixate the index fingertip with both eyes open. In the central patch of the now perceived three, overlapping gratings will begin to compete perceptually.

If portions of each eye’s stimuli are combined with each other, the visual system can also organise a fully coherent percept, a process known as interocular grouping^9–11^. Examples of simple grating stimuli used to generate exclusive, interocularly grouped (IOG) percepts, and actually used in the current study, are seen in Fig. 1.

The effects of physical changes in stimuli on perception for IOG^10–13^ and CBR^14–18^ have been studied intensively. These studies often used achromatic grating stimuli, defined by luminance changes, or stimuli with colour differences.

Recently we reported that CBR can also be generated with orthogonally oriented contrast-modulated (CM) noise gratings, i.e. second-order stimuli, for which there is no change in mean luminance across the stimulus^19^. CM grating stimuli generated much lower proportions of exclusive visibility and a lower number of perceptual alternations during CBR than gratings that were defined by luminance (L) or luminance-modulated dynamic noise (LM), i.e. first-order stimuli, but much higher proportions of “mixed” percepts^19^. In a subsequent study, we found that CM gratings generate greater proportions of superimposition than similarly visible LM stimuli^20^. Along with evidence from other studies^21–23^, we suggested that CM stimuli, unlike L and LM stimuli, are initially processed by binocular neurones. In the current paper, we investigate whether or not CM stimuli generate IOG percepts.

The relationships between physical properties of either one, or both rivalling stimuli and perception during CBR have been described by Levelt's laws^16^ and updated by in later years^24^. The modified four laws are:

I. Increasing stimulus strength for one eye will increase the perceptual predominance of that eye’s stimulus.

II. Increasing the difference in stimulus strength between the two eyes will primarily act to increase the average perceptual dominance duration of the stronger stimulus.

III. Increasing the difference in stimulus strength between the two eyes will reduce the perceptual alternation rate.

IV. Increasing stimulus strength in both eyes while keeping it equal between eyes will generally increase the perceptual alternation rate, but this effect may reverse at near-threshold stimulus strengths. (^24^, page 27).

A recent study showed that IOG perception also depends on stimulus visibility, obeying Levelt’s first three laws when changing colour saturation of unilateral chromatic stimuli^13^. However, during CBR we have shown that none of Levelt’s laws hold when LM stimuli are presented to one eye, and CM stimuli are presented to the other^1^. In the current study, we test whether these laws hold for IOG when using L, LM and CM stimuli. More specific aims are (1) to investigate whether IOG can be generated with CM stimuli and whether perceptual characteristics differ from those found for L and LM stimuli and (2) to test Levelt’s laws using LM and CM stimuli while applying the split-grating IOG paradigm. If IOG is driven by inhibition between low-level monocular processing mechanisms^12,25^, then CM stimuli would not generate IOG percepts, or would show much lower proportions of IOG than L and LM stimuli at comparable visibility. Instead, CM stimuli might demonstrate a predominance for fusion, rather than IOG^20^. If IOG is mediated by later binocular processing mechanisms in agreement with Kovacs et al.^11^, then more IOG events for CM, compared to L and LM stimuli, would be predicted. When IOG across stimulus types is tested, if both types are processed at the same stage, results should be similar and depend on visibility in the same way. If different stages are separately engaged, then different patterns of results would be found.

## Methods

### Participants

#### First experiment: IOG with L, LM and CM stimuli

Eight participants (four males, four females) with an average age of 26 ± 7 years carried out the experiment. All participants had experience in CBR experiments but were naïve to the purpose of the study (except author JS). Prior to the experiments, a vision screening was carried out by an optometrist to ensure normal binocular vision. Specifically, all participants had normal or corrected-to-normal visual acuities of at least 6/6 in both eyes. Far and near cover tests were carried out to ensure that no heterotropia was present. Normal binocular vision was indicated by random-dot-stereopsis of at least 60 arcsec when measured with the Dutch Organization for Applied Scientific Research (TNO) stereo test (Lameris Ootech, Ede, Netherlands).

##### Control experiment: varying CM grating visibility

Three female participants, who did not carry out the main experiment, and one male (author JS) with an average age of 26 ± 4 years, took part in the experiment. All other inclusion criteria were as for the first experiment.

##### Second experiment: testing Levelt’s laws for IOG

Testing 1st, 2nd, and 3rd law: nine participants (four males, five females) with an average age of 27 ± 4 years carried out this part of the experiment. All participants except author JS were naïve to the purpose of the study. All other inclusion criteria were as described as for the first experiment.

Testing 4th law: Four male and five female participants with an average age of 29 ± 4 years, carried out the experiment. Two of those participants did not take part in the first part of the experiment. All other inclusion criteria were as described above.

#### Stimuli

##### First experiment: IOG with L, LM and CM stimuli

Both horizontal and vertical sinusoidal split-gratings were made of a spatial frequency of 2 c/deg within a circular aperture of 2 deg diameter. As shown in Fig. 1, the split occurred in the centre of the stimulus along its horizontal axis. For the left eye’s stimulus, each horizontal split-grating component was above the vertical component for four trials per condition, and for the other four trials below the vertical component (a vice versa arrangement was in the right eye’s stimulus). Both eyes’ stimuli were surrounded by a circular fusion lock with a diameter of 4 deg and a width of 2.6 arcmin (2 pixels).

Estimates of visibility levels of the L and LM gratings were chosen to be above, equal, and below those of the CM gratings based on detection thresholds chosen to compare IOG findings with previous CBR findings^19^. L gratings had a contrast of 0.03, 0.08 and 0.98, which is approximately 3, 8, 98 times above detection threshold. LM gratings were created by adding dynamic two-dimensional binary noise with an amplitude of 0.2 to a sine-wave with luminance modulation of 0.06, 0.10, and 0.78, which is approximately 3, 5, 43 times above detection threshold. The same noise amplitude was multiplied by the sine-wave to create CM gratings with a modulation of 1.00, which is approximately seven times above detection threshold. Dynamic noise was used for CM to avoid any clumping effects that might introduce first order artefacts^26^ and for LM to ensure comparability with CM stimuli. The noise check size was 2 × 2 pixels and each page was displayed for 14.28 ms (two monitor frames with the monitor running at 140 Hz). Stimuli were presented on a grey background with a mean luminance of 50 cd/m^2^. The stimuli were viewed through a stereoscope. The effective viewing distance was 100 cm and the screen pixel size at this distance was 1.3 arcmin.

The stimulus types can be mathematically described as follows.

Sinusoidal luminance (L) grating:

$${l}_{0}\left(x,y\right)={l}_{0}\left[1+l sin\left(2\right.\right.\pi x{f}_{x})]$$

The luminance at position (*x*, *y*) is *l*_*0*_ (*x*, *y*), the mean luminance is *l*_*0*_, and the spatial frequency is *f*_*x*_.

Sinusoidal luminance-modulated (LM) grating:

$${l}_{0}\left(x,y\right)={l}_{0}\left[1+n N\left(x,y\right)+l sin\left(2\right.\right.\pi x{f}_{x})]$$

Two-dimensional binary white noise added to a sinusoidal luminance grating. *N* is the binary noise at position (*x*, *y*) [either black (− 1) or white (1)] and *n* is contrast of 0.2.

Sinusoidal contrast-modulated (CM) grating:

$${l}_{0}\left(x,y\right)={l}_{0}\left[1+n N\left(x,y\right)+nN\left(x,y\right)m sin\left(2\right.\right.\pi x{f}_{x})]$$

Contrast modulation is *m*. The mathematical term $n N\left(x,y\right)m sin(2\pi x{f}_{x})$ expresses the contrast-modulated grating that results from the multiplying random noise sample by a sinusoid^26,27^.

Ten stimulus pages were created using the equations above, each with a different, random noise pattern. These ten pages were cycled in random order for the duration of the trial to generate dynamic noise to avoid consistent first-order artefacts in second-order stimuli due to pixel clumping that would occur if static noise were used^26,28–31^.

##### Control experiment: varying CM grating visibility

Eight trials for CM stimulus modulations of 1.00 and 0.50, resulting in approximately 7 and 3.5 times above detection threshold, respectively, were carried out. All other conditions were the same as in the first experiment.

##### Second experiment: testing Levelt’s laws for IOG

LM-vs-CM conditions: the visibility levels of the LM gratings were chosen to be above, equal, and below that of the CM stimulus. The CM stimulus was fixed at approximately seven times above detection threshold, which is the maximum modulation (1.0) whereas LM contrast was set to 3.5, 7, 43 times above detection threshold (i.e. contrasts of 0.07, 0.14, 0.78, respectively). As for the first experiment, these estimates are based on detection threshold measurements of a previous study^19^. To test the fourth law, both competing gratings were in one condition 3.5 and in another 7 times above threshold. Stimuli were presented on a grey background with a mean luminance of 49 cd/m^2^. All other conditions were the same as for the first experiment.

LM-vs-LM conditions: the visibility levels of the LM gratings were chosen to be above, equal, and below of those of the fixed LM grating of seven times detection threshold, so were 3.5, 7, and 43 times above detection threshold. All other conditions were the same as described for the LM-vs-CM conditions.

#### Apparatus and monitor calibration

A Mitsubishi Diamond Pro 2070SB CRT Monitor with a resolution of 1,027 × 769 pixels was used for stimulus presentation. Dell Precision 3,500 hardware and a customised MatLab program in combination with the Cambridge Research Systems Visual Stimulus Generator (ViSaGe) were used to create and present the stimuli as well as run the experiment. Gamma correction was carried out, using a Cambridge Research Systems ColorCal and software to produce lookup tables, to correct the monitor’s inherent nonlinear luminance intensities after a suitable warm-up period to ensure a consistent mean luminance. A four-mirror stereoscope composed of optical components by OptoSigma (OptoSigma Corporation, California, USA) was used for viewing stimuli. The mirrors were carefully aligned prior to experiments to ensure that only one stimulus was visible to each eye and to ensure that ocular alignment was comfortable.

#### Procedure

Written and verbal information about the project was provided in advance to the participants and they gave written informed consent before taking part. Ethics approval to conduct the experiments on human participants was in line with the ethical principles of the Helsinki declaration of 1975 and conduct of the project was approved by the Faculty of Science and Technology Research Ethics Panel (FST/FREP/12/327) at Anglia Ruskin University. Participants were reimbursed for time spent. All experiments were performed in a dark room. Participants sat on a comfortable chair and placed their heads in a chin- and forehead rest. Before an experiment for a participant began, the stimuli were aligned by adjusting the position of a left and right nonius markers on the screen to ensure comfortable viewing with both eyes. The task was to indicate via pressing and holding button (A) for an exclusively horizontal IOG, button (B) for an exclusively vertical IOG, and both buttons A and B for piecemeal and/or eye-of-origin percepts (referred as ‘other percepts’). When no buttons were pressed, superimposition was perceived. The participants were instructed to fixate the centres of the gratings.

##### First experiment: IOG with L, LM and CM stimuli

Two experimental sessions, each on a different day, were carried out. Each session included four repeats of the following order of trials: L 98 (i.e. luminance-defined stimuli with approximately 98 times above detection threshold based on mean findings of a previous study^19^), L 8, L 3, LM 43, LM 5, LM 3 and CM 7. Results from this previous study showed inter-individual variability in mean detection thresholds of ± 7% (across five participants) for both first- and second-order stimuli. Each trial lasted at least 120 s. Instructions and practice trials were given before formal data collection started. Breaks between trials were permitted whenever desired. A complete session lasted between 55 and 75 min, depending on the breaks for each participant.

##### Second experiment: testing Levelt’s laws for IOG

Three experimental sessions were carried out. The first and second session included the testing of the first three laws for both LM-vs-CM and LM-vs-LM conditions, respectively. These sessions included eight repeats of the following order of trials: 3.5 vs 7, 7 vs 7, and 43 vs 7 approximately times above thresholds based on results from a previous study^19^. Orientation and stimulus type location were counterbalanced between trials. Each trial lasted at least 120 s. Instructions and practice trials were given before formal data collection started. Breaks between trials were permitted whenever desired. A complete session lasted between 50 and 60 min, depending on the breaks for each participant. The third session tested the fourth law for both LM-vs-CM and LM-vs-LM condition. For each condition, 16 trials were carried out, so the third session took 60–80 min.

#### Data analysis

##### First experiment: IOG with L, LM and CM stimuli

The total perceptual durations of IOG events (i.e. sum of responses for a horizontal IOG and the vertical IOG percept), superimposition, and the other percepts across 120 s and their respective mean durations were calculated. Perceptual changes were measured during each trial and averaged for each condition across trials and participants. Full flips were defined as changes from one exclusive percept to another (without any superimposed or other percept between). All other possible perceptual changes were defined as half flips.

Very short response durations (≤ 180 ms) were excluded to avoid responses that are unlikely due to a fast reaction of a participant but rather due to a not completely pressed response-box button^19^. A customized Matlab program was used to analyse the raw data generated from this study. Repeated measures ANOVAs with Greenhouse–Geisser correction and if significance was found, planned comparisons were carried out using Statistica (Stat Soft, Int., USA).

##### Second experiment: testing Levelt’s laws for IOG

Total perceptual durations of IOG events and their means, as well as the numbers of full flips were averaged across trials and participants. All other parts of the analysis were performed as in the first experiment.

##### Analysis of perceptual phase distributions

The distributions of IOG phase durations were fitted with a gamma function using Matlab. For each condition and each subject, data was first normalized by dividing the phase durations by the relevant mean. These normalised data were then combined across subjects. The perceptual phases are presented in the following form using a gamma distribution:

$$f\left(x|\alpha ,\beta \right)=\frac{1}{{\beta }^{\alpha }\Gamma (\alpha )}{x}^{\alpha -1}{e}^{\frac{-x}{\beta }};x>0,\alpha >0,\beta \ge 0$$

The gamma function is indicated with Γ $(\alpha$), the “shape” parameter is $\alpha$ and represents the skewness of the distribution, the “scale” parameter $\beta$ scales the distribution along the abscissa, and the number of perceptual events is *x*^16,32,33^. The coefficient of determination *R*^*2*^ has been used in previous studies^33–35^ as an indicator of how well actual data fit a predicted model; the closer *R*^*2*^ is to 1, the better fit of the model to the actual data.

## Results of first experiment: IOG with L, LM and CM stimuli

In the following section, we calculated typical CBR and IOG parameters that have been used to describe their perceptual dynamics^11,14–16,36^. Specifically, we report relative and mean durations of the various perceptual states, the various types of alternation (or flips) between different states, and the distribution of the exclusive visible events.

The main aim of the current experiment was to test whether CM (second-order) stimuli undergo inter-ocular grouping (IOG) in a similar way to similarly visible L and LM (first-order) stimuli. Data were analysed according to stimulus type (L, LM and CM), stimulus visibility (low, medium, high) and perceptual state (exclusive visibility/IOG, “other states” of piecemeal/eye of origin, and superimposition). Dependent measures were relative proportions (%) and mean durations (s). As there were different numbers of visibility levels for L and LM (low, medium, high), versus CM (medium) stimuli, a repeated measures ANOVA analysis was first used to compare results for similarly visible L, LM and CM stimulus types, and then a separate analysis was used to compare across visibility levels for the first-order stimulus type (so L and LM only). Planned comparisons were subsequently used to test individual means across types and visibility levels within each analysis. Independent two-tailed t-tests compared means between analyses. Detailed statistical results including additional planned comparisons not mentioned in text for improved readability, can be found in the Supplementary Information.

### Perceptual states and mean durations

When testing across similarly-visible stimulus types (L, LM, CM) using relative measures (%) for IOG, superimposition or “other states”, there was no main effect of stimulus type, but the effect of stimulus type depended strongly on the perceptual state measured, as revealed by the significant interaction [F(1.9, 13.4) = 23.0, p < 0.0001]. Planned comparisons revealed that all types were significantly different from each other for all states, but perceptual states for CM stimuli, showed greater differences than did LM and L stimuli (see Supplementary Information). In other words, when results of first order stimuli (L and LM) are compared with those for second-order stimuli (CM), differences are greater than those found between noiseless (L) and noisy (LM) stimuli.

**Figure 2:**
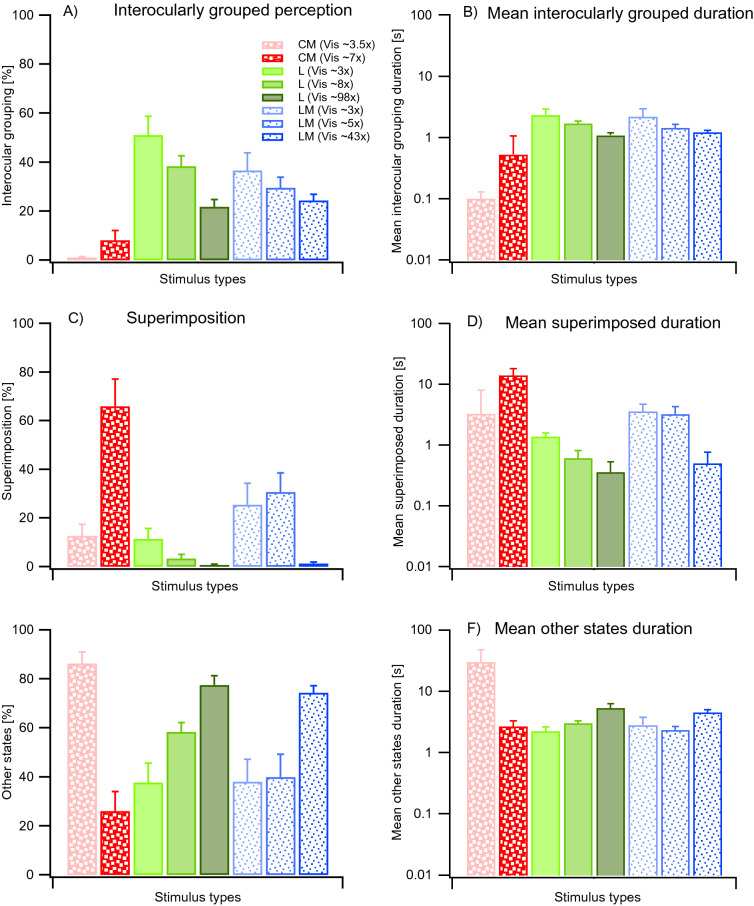
Results for the relative proportions of interocular grouping (**A**), superimposed perception (**C**) and all other states (**E**) and their respective mean durations (**B**), (**D**) and (**F**) using CM, L, and LM stimuli, averaged across trials and participants. Red bars with big dots represent CM-stimuli, blue bars with small dots LM-stimuli, green bars L stimuli. Visibility (Vis) refers to approximated times above detection threshold (see also “Stimuli”). Depicted is also CM Vis 3.5× data from the control experiment. Error bars indicate + 1 SEM.

As shown in Fig. 2A, CM IOG percepts for Vis 7× occurred only sporadically or not at all (8.1 ± 4.0% standard error (SE), averaged across trials and participants) compared to similarly visible L (38.3 ± 4.2% for Vis 8×) and LM (29.5 ± 4.4% for Vis 5×) stimuli. CM stimuli generated predominantly superimposition (66.0 ± 11.2%) whereas similarly visible L (3.3 ± 1.8%) and LM stimuli (31 ± 8%) did not (Fig. 2C). With regards to “other states” (see Fig. 2E), CM stimuli generated fewer proportions (at 26.0 ± 8.0%) than L (58.4 ± 3.9%) [F(1, 7) = 34.78, p = 0.0006; planned comparison] and LM stimuli (39.9 ± 9.3%) [F(1, 7) = 10.47, p = 0.014; planned comparison].

In the control experiment, in which two different visibility CM stimuli are tested for 4 participants (data for approximately 3.5× visibility are appended to Fig. 2), lowering CM visibility from approximately 7–3.5 times threshold led to a decrease in superimposition (7×: 49.1 ± 20.9% and 3.5×: 12.7 ± 4.7%). IOG rarely occurred (7×: 2.5 ± 1.6% and 3.5×: 1.1 ± 0.3%). Proportions of “other states” for CM stimuli increased with decreasing visibility (7×: 48.3 ± 19.5 SE and 3.5×: 86.3 ± 4.7%).

The separate analysis of L and LM stimuli across different visibility stimuli revealed a significant main effect of visibility on relative proportions of perceptual state, and an interaction between stimulus type, visibility, and perceptual state. The subsequent planned comparisons can be found in the Supplementary Information.

Regarding the mean durations, there were significant main effects of stimulus type [F(1.1, 7.8) = 8.5, p < 0.05] and perceptual state F(1.1, 7.4) = 6.2, p < 0.05], although a significant interaction was found between them [F(1.1, 7.9) = 9.3, p < 0.05]. Planned comparisons revealed significant differences (at p < 0.05) between first and second order stimuli in terms of the exclusive visibility (IOG) percept for which it was significantly shorter for second-order, than for first-order stimuli, and for superimposition, it was significantly longer for second-order, than for first-order stimuli. The shorter mean durations for CM IOG (0.6 ± 0.2 s) compared to L (1.7 ± 0.2 s) and LM (1.4 ± 0.2 s) stimuli are shown in Fig. 2B. The results of the effects for lowering visibility on grouping for CM stimuli (to Vis 3.5×) are appended to Fig. 2. However, these were obtained in a control experiment and are mentioned separately in a paragraph below.

The mean durations of superimposition (Fig. 2D) were longer for CM Vis7× (14.0 ± 4.2 s) compared to similarly visible L (0.6 ± 0.2 s) and LM (3.2 ± 1.0 s) stimuli. The mean durations for other percepts did not differ significantly when comparing first-order (L 3.0 ± 0.3 s and LM 2.3 ± 0.3 s) and second-order stimulus results (CM 2.7 ± 0.6 s).

When comparing first-order stimulus types (i.e. L versus LM) across different visibility levels and perceptual states, the perceptual state was significant [F(1.2, 8.5) = 1.3, p < 0.01], as was the interaction between type and visibility [F(1.1, 7.9) = 6.6, p < 0.05], and between type and state [F(1.9, 13.5) = 10.6, p < 0.01] (see Supplementary Information).

### Perceptual flips

Full- and half-flip rates were statistically compared in a similar way to relative proportions and mean durations above. For similarly visible L, LM and CM stimulus types significant main effects were found for stimulus type [F(1.9, 13.2) = 50.8, p < 0.0001] and flip type [F(1, 7) = 50.0, p < 0.001], but they also interacted [F(1.5, 10.6) = 74.9, p < 0.0001]. This was due to significantly more half- than full-flips being found for all stimulus types, with significantly different rates being found between the first- and second-order half-flips (see Supplementary Information).

**Figure 3:**
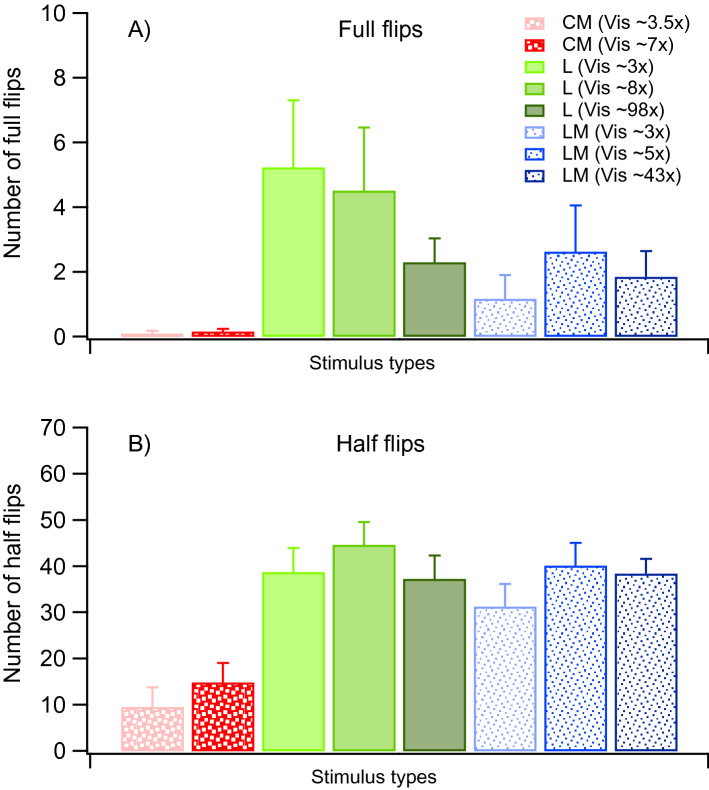
Results for number of full flips (**A**) and half flips (**B**) averaged across trials and participants. Red bars with big dots represent CM-stimuli, blue bars with small dots LM-stimuli, green bars L stimuli. Visibility (Vis) refers to the approximated times above detection threshold of a stimulus type (see also “Stimuli”). Depicted is also CM Vis 3.5× data from the control experiment. Error bars indicate + 1SEM.

Full-flips were defined as changes from one exclusive percept to another without any superimposition or other percept between. As shown in Fig. 3A, CM stimuli resulted in no such alternations (0.2 ± 0.1 full flips/trial), whereas comparable visibility L (Vis 8×; 4.5 ± 2.0 full flips/trial) and LM (Vis 5×; 2.6 ± 1.4 full flips/trial) stimuli showed more flips. An interaction between visibility and stimulus type was found to be significant [F(1.5, 10.6) = 74.9, p < 0.0001]. Planned comparisons showed however that no statistical differences between stimulus types were reached [p > 0.05; see also Sect. 1.3 in Supplementary Information].

Half-flips were defined as all flips that were not full flips (Fig. 3B). For all stimulus types, more half- than full-flips occurred. CM generated significantly fewer half-flips (Vis 8×; 14.8 ± 4.3 half-flips/trial) than comparable visibility L (Vis 8×; 44.7 ± 3.3 half-flips/trial) and LM (Vis 5×; 40.2 ± 4.9 half-flips/trial) stimuli [both planned comparisons: p < 0.0001].

When comparing first-order stimulus types, visibility, and flip type, main effects of stimulus type [F(1, 7) = 12.8, p < 0.01] and flip type [F(1, 7) = 72.0, p < 0.0001] were found, which was due to more half than full-flips and a greater number of alternations for L, compared to LM stimuli.

When lowering CM’s visibility, the average number of half-flips lowered from 16.7 ± 12.1 half-flips/trial to 9.6 ± 5.4 half flips/trial (~ Vis 7× to 3.5× threshold), a trend which did not reach statistical significance when testing for main effects of visibility [F(1, 3) = 1.1, p = 0.37] and flip type [F(1, 3) = 2.3, p = 0.23] (see supplementary Information).

### Distribution of interocularly grouped phases

**Figure 4:**
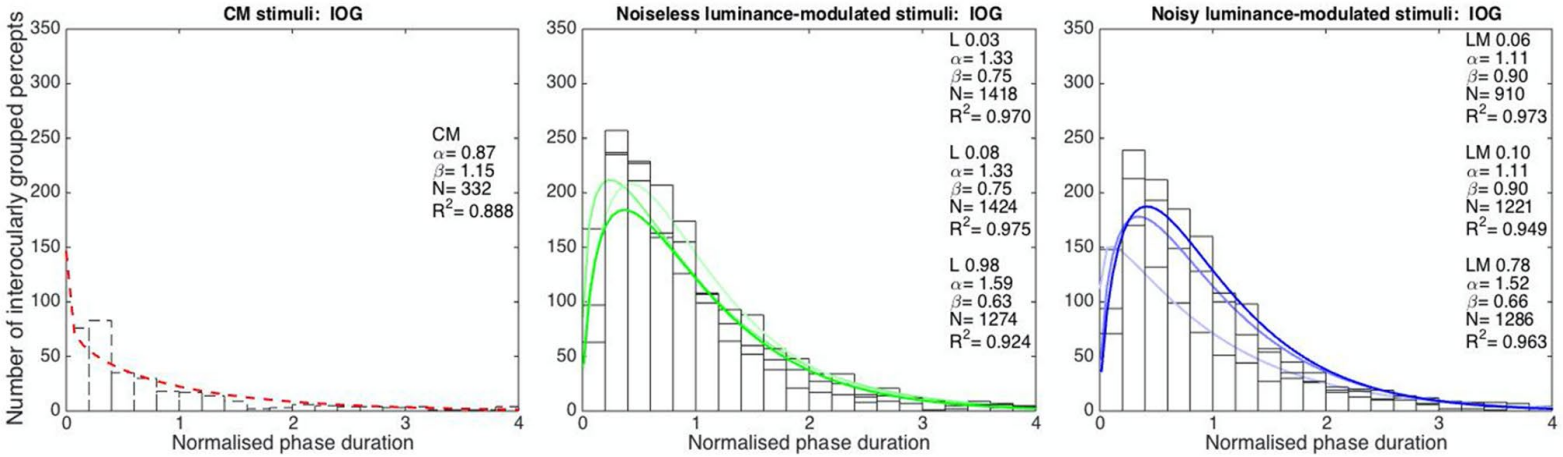
Gamma functions fit to numbers of interocularly grouped CM (left), L (middle) and LM (right) percepts. Different visibility levels for L and LM stimuli are represented by different colour contrasts (e.g. LM 0.78 is represented in darkest blue while LM 0.06 lightest blue). The x-axes depict normalized phase durations. The y-axes represent the number of perceptual events. The fitted gamma functions are shown together with parameters for CM (red), LM (blue) and L (green) stimuli. Please note that CM Vis 7× is depicted only as CM Vis 3.5× generated almost no IOG percepts for the four participants taking part in the control experiment.

Both L and LM phase distributions for interocularly grouped percepts show a typical gamma-shape (Fig. 4), known as a hallmark of CBR^16,37^, which has also been shown for IOG^11^. CM stimuli generated fewer interocular grouped events. The coefficient of determination indicated by R^2^ values is closer to 1 for L and LM stimuli than for CM stimuli.

### Comparison of superimposition: CBR and IOG paradigm

**Figure 5:**
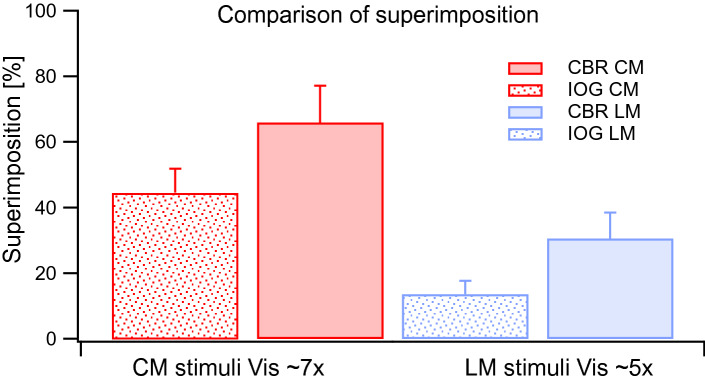
Comparison of superimposition during a CBR^20^ (shaded bars) and current IOG results (dotted bars), i.e. results from the first experiment of this paper). CM is indicated as red and LM as blue. Error bars indicate + 1 SEM.

We also compared the relative proportions of superimposition with data taken from an earlier study^20^ generated by a CBR paradigm with otherwise comparable stimulus properties (Fig. 5). CM stimuli generate greater proportions of superimposition compared to LM stimuli when either the IOG or CBR paradigm is used. Greater proportions of superimposition are also apparent for CBR compared to IOG. To compare superimposition means between CM CBR and CM IOG as well as LM CBR and LM IOG, independent two-tailed t-tests were performed, which found that these differences were not statistically significant for CM [t(21) = 1.52, p = 0.17] or LM conditions [t(21) = 2.13, p = 0.16].

## Results of second experiment: testing Levelt’s laws for IOG

Experiment 2 aimed to test Levelt’s laws when using split-gratings made of both LM and CM components in an IOG paradigm for which the visibility of the LM component was changed relative to the CM component (the maximum visibility for CM stimuli is ~ 7× threshold^19^). The results were compared to IOG for different visibility LM-LM split gratings. Due to the paucity of IOG percepts generated for CM-CM split grating stimuli (see Fig. 2A), we did not investigate Levelt’s laws for them. Due to our findings for CBR^1^, we hypothesized that for LM–CM split gratings, IOG would not be in line with Levelt’s laws.

### Levelt’s first law

The main effects of the stimulus type for both CM-vs-LM and LM-vs-LM experiments were separately analysed as were exclusive visibility results for the three different visibility conditions. Planned comparisons were used to test differences between stimulus type and visibility conditions. As the same stimulus type was used for the LM-vs-LM, we refer here to ‘Eye’ (i.e. fixed vs. variable), rather than the stimulus type.

Levelt’s first law predicts that a grating of higher visibility will predominate perception (Fig. 7A).

#### LM-vs-CM experiment

**Figure 6:**
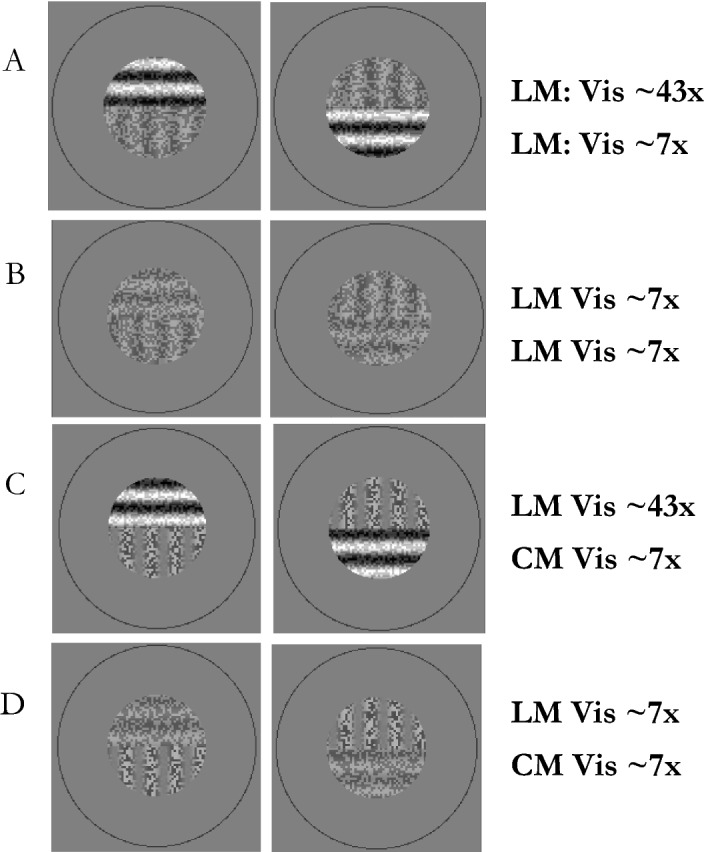
Example stimuli used in Experiment 2. Vis refers to the estimates of multiples above detection threshold. (**A**, **B**) Show horizontally split LM gratings. (**C**, **D**) Show horizontally split LM and CM gratings.

**Figure 7:**
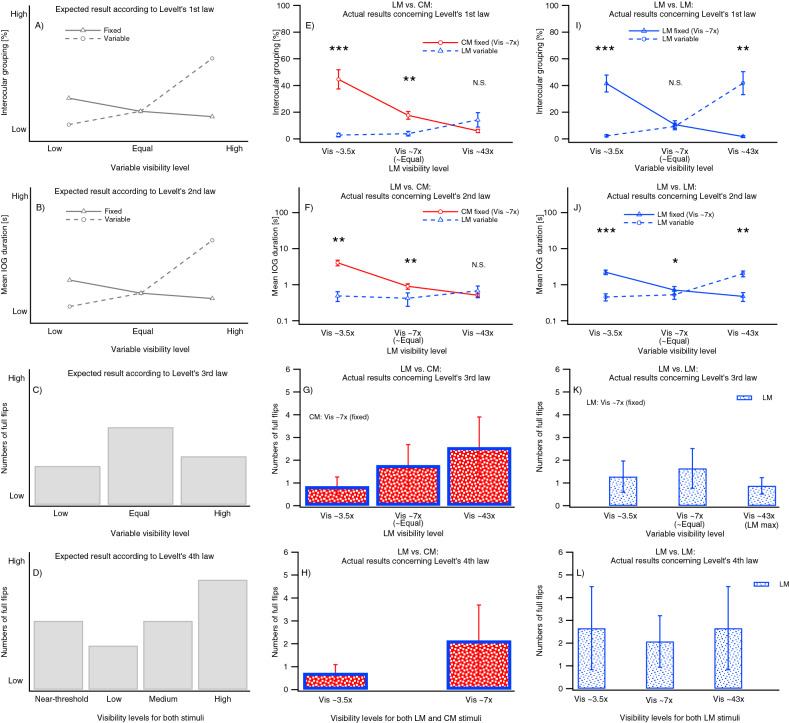
(**A**–**D**) Predictions following Levelt’s four laws, (**E**–**H**) LM-vs-CM conditions, (**I**–**L**) LM-vs-LM stimuli conditions. N.S. means not significantly different and the asterix refers to a significant difference (i.e. asterix symbols: *p ≤ 0.05; **p ≤ 0.01; ***p ≤ 0.001). The error bars indicate ± 1 SEM.

Counterintuitively and at the same time the highlight of the current study is the result that when split-gratings with a high-visibility LM and a low-visibility CM component are presented to the eyes (see example in Fig. 6C), the proportion of time for IOG of the LM grating was never greater than for the CM grating (Fig. 7E).

An interaction between visibility level and stimulus type was found [F(1.2, 9.3) = 17.3, p = 0.001]. Planned comparisons revealed significantly greater proportions of CM IOG than LM IOG [F(1, 8) = 22.0, p = 0.0016] when using similar visibility level gratings (LM ~ 7× vs. CM ~ 7×) and for the LM ~ 3.5× vs. CM ~ 7× [F(1, 8) = 26.4, p > 0.001]. There was no difference in proportions of IOG for LM and CM gratings when visibilities were for LM ~ 43×, and for CM ~ 7× [F(1, 8) = 2.1, p = 0.18].

#### LM-vs-LM experiment

The results for competing LM-vs-LM split-gratings (see examples in Fig. 6A,B) follow the predicted pattern according to Levelt’s first law (Fig. 7I). An interaction between visibility level and ‘eye’ was found [F(1.1, 8.6) = 24.8 p = 0.0007] with significant differences [p < 0.01; planned comparisons] between all except the LM ~ 7× vs. CM ~ 7× condition (see Supplementary Information).

### Levelt’s second law

The second law predicts that mean exclusivity duration of the stronger stimulus will increase as the difference in visibility between rivalry stimuli increases, whereas mean exclusivity duration of the weaker stimulus will decrease slightly (Fig. 7B). The same statistical protocol as for the first law was applied.

#### LM-vs-CM experiment

When split-gratings of high-visibility LM and low-visibility CM components are presented, the prediction of the second law fails (Fig. 7F depicted without outlier values of two participants). A near-significant interaction between type and visibility level was found when all participants were included [F(1, 8.2) = 43.3, p = 0.069]. When two extreme outlier values were excluded from the analysis, the interaction was highly significant [F(1.1, 6.8) = 25.3, p = 0.001]. Planned comparisons (see further analysis Sect. 2.2 in Supplementary Information) revealed significant differences between all but the LM43× vs. CM 7× condition, highlighting the key finding of the present study.

#### LM-vs-LM experiment

The results for LM-vs-LM split-gratings do follow the pattern as predicted by Levelt’s second law (Fig. 7J). When excluding outliers from the data, an interaction between visibility and flip type is found [F(1.0, 6.0) = 31.5, p = 0.0004]. Planned comparisons were significantly different, with the weakest effect found for the LM 7× vs LM 7× experiment (see further analysis Sect. 2.2 in Supplementary Information).

### Levelt’s third law

The third law predicts that the full flip rate should be highest when visibility levels of two rivaling stimuli are the same. Full flip rate should reduce as the difference between the visibility levels increases (Fig. 7C). A similar statistical analysis was carried out for the CM-vs-LM and for LM-vs-LM conditions to test the effect of visibility change on the full flip rate. However, the traditional assumption of complete alternations without mixed states in-between, i.e. full flips, might be too strict a criterion. Hence, we also tested the effect of unilateral visibility change on the half flip rates (see Supplementary Information).

#### LM-vs-CM experiment

The results for numbers of full flips do not follow this prediction when CM and LM gratings compete (Fig. 7G). Increasing the visibility level difference between stimuli increased the full flip rate and did not show the predicted inverted U-shape. No main effect of visibility was found for full flips [F(1.3, 10.8) = 2.5, p = 0.14]. They occur most often when the difference in visibility between LM and CM components was greatest (2.6 ± 1.3 full flips/trial). They occur less often as the difference in visibility decreases. For approximately equal visibility gratings (7× vs 7×; see example Fig. 6D) there are 1.8 ± 0.9 full flips/trial. The same increasing trend was observed (however not depicted in Fig. 7) when using half flips, where the main effect was significant [F(1.8, 14.3) = 5.1, p < 0.05].

#### LM-vs-LM experiment

The pattern in Fig. 7K tend to follow the prediction of Levelt’s third law, although there was neither a statistically significant main effect of visibility on numbers of full-flips [F(1.4, 11.4) = 1.6, p = 0.24], nor half flips [F(1.1, 9.1) = 0.9, p = 0.39] (not shown).

### Levelt’s fourth law

The fourth law predicts that the flip rate should increase when increasing the visibility levels of two rivalling, but equally visible stimuli, although this trend might reverse near threshold (Fig. 7D).

Separate repeated measures ANOVAs were performed to investigate the effects of bilateral changes of visibility on full- and half-flips for the CM-vs-LM and for the LM-vs-LM experiments.

#### LM-vs-CM experiment

The number of full-flips increased slightly with changes in visibility (0.7 ± 0.4 full flips/trial for 3.5× vs. 3.5× condition to 2.2 ± 1.5 full flips/trial for 7× vs. 7× condition), but did not reach statistical significance for full- [F(1, 8) = 1.3, p = 0.29] (Fig. 7H) or half-flips [F(1, 8) = 0.1, p = 0.77] (see Supplementary Information).

#### LM-vs-LM experiment

There were no significant main effects of visibility on measured number of full- [F(1.4, 10.8) = 0.1, p = 0.82] (Fig. 7L) or half-flips [F(1.4, 11.2) = 1.0, p = 0.38] (see Supplementary Information). The fourth law leaves room for interpretation about whether the data are in line with the prediction. The trend shown in Fig. 7L could be in line with the modified 4th law, assuming 3.5× is “near-threshold”.

## Discussion

### IOG percepts are rare for the CM-vs-CM condition

One of the key findings of the current study is that the second-order only (i.e. CM-vs-CM) condition initiated very few and brief IOG events but significantly more superimposition, when compared to comparable first-order, LM stimuli (Fig. 2). IOG proportions were lower but not significantly different from proportions of exclusivity during CBR found in our previous study (Fig. 5).

Given the constant stimulus order and the prolonged testing duration for the first experimental sessions, one possibility is that a cumulative effect across trials might have been observed as longer exposure to rivalrous settings may alter proportions of mixed perceptual states^38^. We think that this is not likely to have influenced our results as frequent breaks between relatively short, 2-min trials were encouraged outside the stereoscope with normal binocular viewing throughout each session. Mean results from trials over time were also analyzed using repeated measures ANOVAs to check this possibility. There was no overall effect of trial number [F(1, 7) = 2.0, p = 0.20] or session number [F(1, 7) = 0.2, p = 0.68] on our results, nor did they vary depending on stimulus type or perceptual category (see Sect. 3.0 in Supplementary Information).

CM stimuli are thought to be processed by a filter-rectify-filter pathway^28,39^. While the first stage processes the carrier noise information of the CM grating (the carrier processing stage), the second stage decodes the grating (the envelope processing stage). Envelope processing neurones are likely to be orientation-selective, given that in area V1 and V2, neurones demonstrate orientation tuning to CM stimuli^40,41^. It has also been suggested that the neurones at the second CM processing stage are in an area that is substantially binocular^21,23,42,43^ possibly beyond V1, for example in V2, where disparity processing neurones predominate^44^. Binocular neurones in V2 of strabismic (hence non-binocular) monkeys show reduced responses^45^. In one human neuro-imaging study^46^ and in electrophysiological studies on cat^47^ and monkey^41,48^, greater activity is found in V2 when using CM, compared to LM stimuli. Other psychophysical studies report results that support a binocular site for the second stage that enables extraction of CM envelopes^21–23,43,49^ and we published data concerning CBR for a CM-vs-CM stimulus condition, finding that significantly higher proportions of superimposition occur, than for equally visible LM-vs-LM gratings^20^. In the current study we extend these investigations to IOG.

The results of our first experiment using CM-vs-CM split gratings show that no significant IOG occurs but instead, significant proportions of superimposition are found compared to when LM-vs-LM split gratings are viewed (Fig. 2). Superimposition during CBR using standard luminance (L) gratings has been suggested to be the result of binocular fusion^7,8^. Hence, we suggest that high proportions of superimposition during IOG using CM-vs-CM split gratings (Fig. 2), like in CBR^19,20^ are due to envelope processing neurones that do not engage in mutual inhibition, but rather in binocular fusion. The observed results might occur for IOG based on a framework similar that we proposed for CBR for L, LM, and CM gratings^1^, which includes mutual inhibition model like proposed for CBR^50,51^, the presence of orientation-selective neurones within ocular dominance columns^12^ and the filter-rectify-filter model for CM stimuli^26,52^. For both CBR and IOG, we show that rivalrous orthogonally oriented CM gratings generate only low amounts of exclusive visibility and few alternations, which might demonstrate that the second processing stage engages in minimal mutual inhibition and that intrinsic noise causes the alternations observed^19,20^. We did not investigate Levelt’s laws with CM-vs-CM split gratings in the current study because almost no IOG percepts were generated and therefore very few perceptual alternations.

### Do Levelt’s laws hold for IOG?

Levelt’s first three laws apply to IOG when using unilateral changes of colour saturation of chromatic stimuli^13^. Levelt’s fourth law was not tested. Table 1 compares these previous results with findings of the current study. For LM-vs-LM split-grating stimuli, IOG characteristics again follow Levelt’s first three laws as they do for CBR^1^. However, when LM-vs-CM stimuli compete for IOG, like CBR^1^, Levelt’s laws do not hold. In terms of Levelt’s 4th law, results are inconclusive for LM-vs-LM and LM-vs-CM stimuli for IOG and it was not tested for L-vs-L stimuli.

**Table 1:** Comparison of whether Levelt’s modified four laws hold for IOG experiments for various stimulus types.

Levelt’s laws	L-vs-L^13^	LM-vs-LM (Results of this paper)	LM-vs-CM (Results of this paper)
First	✓	✓	×
Second	✓	✓	×
Third	✓	×	×
Fourth	–	?	?

The check marks indicate that the laws hold for the specific conditions, the × indicate that the law does not hold, the question marks indicate uncertainty and the hyphen indicates an untested condition.

*L* noiseless luminance modulated (chromatic and achromatic) stimuli.

#### Why do high-visibility LM percepts not predominate over low-visibility CM percepts during IOG?

When high- and low-visibility LM-vs-LM split-gratings are dichoptically presented to the eyes (as shown in Fig. 6C), Levelt’s first law would suggest that the IOG percept is predominately driven by the high-visibility portions (from data of Fig. 7I). Each half of these same orientation high visibility portions are represented in different hemispheres of V1 but each will compete with lower visibility stimuli of orthogonal orientation in the same cortical area. Orthogonally orientation-selective monocular neurones in the same cortical area will mutually inhibit one another via interneurons^51^, so that the stimulus with higher visibility will predominate perception. Grouping across hemispheres results in a predominant percept of a complete horizontally orientated IOG grating. Perception of IOG is not stable due to intrinsic noise (i.e. neuron transmitter release fluctuations and spiking variations^53^), adaptation of neurones^54^, and external noise (i.e. blinks and eye movements), resulting in changes in perception less frequently to the lower visibility IOG, vertical grating.

The main findings of the current study depend on estimating visibility levels based on detection thresholds measured in a previous study^19^ for L, LM and CM grating stimuli (with n = 5 binocularly normal adult participants). We did not measure thresholds for each individual participant in this study. The inter-individual variability across five participants was ± 7% for both first- and second-order stimuli. Participants in both studies had visual acuities in both R and L eyes of 6/6 or better and stereo acuities of 60 arcsec or better and were all young adults (age 26 ± 6 years). The anticipated variance in the current study could reasonably be assumed to be similar and this small variance would not have influenced our outcomes.

We suggested a framework for CBR with a second stage to (1) incorporate the filter-rectify-filter model of processing CM envelopes^39^ and (2) to address counterintuitive findings found for CBR in which high visibility LM gratings never predominated over low visibility CM gratings presented to each eye (Fig. 3; ^1^). In the current study, we find a similar result for IOG using split gratings and this framework again is useful to explain it. The finding is that like in CBR, in IOG high visibility LM gratings do not predominate perception over lower visibility CM gratings (Fig. 7E,F). Orientation-selective monocular neurones that process the first stage of CM stimuli, i.e. the carrier noise, potentially compete with those processing carrier noise from the LM stimulus due to different orientation gratings being constructed, and with luminance gratings at the first-filter stage (as the noise is broad-band). If perception depended only on outputs at this stage, perception of the LM stimulus would predominate over that of the CM stimulus. However, this was not what we found (Fig. 7). We propose that second-stage neurons are inherently binocular, that they extract the CM grating without engaging in interocular competition, and that they instead engage in fusion, reducing the inhibitory advantage of the high-visibility LM portion. Feedback from the second to the first stage occurs due to visual saliency, a low-level process arising as early as V1 (e.g. Li ^55^). Saliency of suppressed images has been shown to dictate the spatial origin of a perceptual change for luminance stimuli during CBR^56^ and so might cause longer durations of predominance for the more strongly suppressed CM, over LM gratings^49^. We also incorporated asymmetric feedback^57^ to reduce the effects of high visibility LM stimuli from the other eye generating similar proportions of IOG percepts to CM gratings (Fig. 7E). An alternative explanation is that envelope processing neurones engage in low levels of mutual inhibition.

A remaining question is, what causes IOG? Kovács et al.^11^ proposed that IOG is essentially a result of a binocular mechanism. Later studies provided evidence for a major role of low-level monocular neurones^12,25^. Our results suggest that low-level IOG can occur primarily with monocularly competing neurones. CM-vs-CM split gratings generate almost no IOG percepts, but generate mainly superimposition and LM-vs-LM split-gratings, which are unlikely to require high level processes, do generate IOG percepts^12,25^. Opponency neurones, a specialised type of neurone that is found in the early visual cortex may mediate asymmetric feedback and visual saliency. Said and Heeger^58^ introduced these neurones to CBR neural computation and suggested that opponency neurones, which receive excitatory input from one eye and inhibitory input from the other eye for each orientation, compute differences between the signals from the two eyes via feedback. Feedback could explain intra- and interhemispheric combination of information, resulting in coherent, exclusive IOG.

For our stimuli, IOG could have occurred both intra-hemispherically or inter-hemispherically (as split gratings occurred along the horizontal midline). Our results are similar to those that we obtained for CBR, which also could have occurred intra-hemispherically or inter-hemispherically. It has been noted previously that hemispherical differences do influence the formation of an IOG percept^12^, but we cannot address these differences with our results. These authors demonstrated that grouping can occur within a hemifield (intrahemispheric), but that it is stronger when luminance-defined gratings are shown to different hemifields (interhemispheric). They speculated that this might be due to stronger “…connections between different eyes [that] are more inhibitory (leading to less grouping) within a hemisphere, compared to between hemispheres.” (^2^, page 7). The question about why opponency neurones have evolved is still unclear. In the light of the current findings and data showing that the visual system tries to form an exclusive visible percept for CBR, opponency neurones may have evolved to facilitate coherent perception when dissimilar monocular inputs causing rivalrous mutual inhibition and potentially mixed perception. Coherent images (here IOG percepts) provide an advantage to navigate in the environment compared to an ambiguous, dissimilar perception.

### Is the process that mediates interocular grouping related to that which generates superimposition?

One recent study showed that when decreasing stimulus strength bilaterally for both split-gratings, IOG increases^59^, which we also found when lowering the contrast bilaterally for L and LM gratings (Fig. 2A). Lowering contrasts of stimuli for both eyes may reduce mutual inhibition and promote binocular combination, i.e. superimposition. This was also observed for CBR using luminance-defined stimuli^7,8^ and for IOG in the current study (Fig. 2C,D). Figure 5 compares proportions of superimposition for CBR and IOG for LM and CM stimuli. CM produced overall more superimposition than LM stimuli for both paradigms. Although IOG generated lower proportions of superimposition in comparison to CBR, this difference was not statistically significant. Studies on CBR have also demonstrated that lowering of contrast, lowers exclusivity but increases perception of superimposition^7,8^. We report for the first time data concerning superimposition using an IOG paradigm. We show that lowering contrast bilaterally increases perception of superimposition for L and LM stimuli (Fig. 2C). This finding could indicate involvement of the same binocular mechanisms for both IOG and superimposition, or simply be a correlation with no causal link.

## Conclusions

We investigated IOG using LM and CM noise gratings, as well as tested Levelt’s laws for IOG. We demonstrate that CM split-gratings almost never form an IOG percept, but generate predominantly superimposition percepts. As for CBR, Levelt’s laws do not hold for IOG across CM and LM stimulus types. This result implies different processing stages for LM and CM stimuli, in line with our conceptual framework established in a study concerning those stimulus types and CBR^1^. It suggests that either visual saliency, asymmetric feedback from the CM second- to the LM first-stage of processing, or a combination of both, contributes to the perceptual outcomes for IOG as well as CBR. We suggest that both CBR and IOG are efforts by the visual system to combine information to form a coherent impression.

## Supplementary information

Supplementary information.
