# Supplementary material for: Contrast-modulated stimuli produce more superimposition and predominate perception when competing with comparable luminance-modulated stimuli during interocular grouping
Source: Sci Rep. 2020 Aug 7;10:13409. doi: 10.1038/s41598-020-69527-5 (PMC7414227; doi:10.1038/s41598-020-69527-5)
Supplement: Supplementary file 1 — Supplementary information. [file 41598_2020_69527_MOESM1_ESM.docx]

**Appendix: Contrast-modulated stimuli produce more superimposition and predominate perception when competing with comparable luminance-modulated stimuli during interocular grouping**

Jan Skerswetat* ^1,2^, Monika A. Formankiewicz ^1^ & Sarah J. Waugh ^1^

^1^ Anglia Vision Research, Department of Vision and Hearing Sciences, Anglia Ruskin University, East Road, CB1 1PT, Cambridge, United Kingdom of Great Britain

^2^ Department of Psychology, Northeastern University, 360 Huntington Ave, Boston, Massachusetts, 02115, United States of America

Corresponding Author *

Email address: [j.skerswetat@northeastern.edu](mailto:j.skerswetat@northeastern.edu)

1. **First experiment: Statistical results for bilateral CM, L, and LM stimulus conditions using an IOG paradigm**

Statistical outcomes for contrast-modulated noise (CM), luminance-modulated noise (LM) and noiseless luminance (L) grating stimuli for the first and second experiments are detailed below. Results are subdivided into an analysis of perceptual states: exclusive visibility/IOG (EV), “other states”, which is either piecemeal (PM) or eye of origin, and superimposition (SI) for the dependent measures of relative proportions (unit: %) and mean durations (unit: seconds). Different perceptual alternation types, namely full and half flips (unit: flips per trial duration) were also analyzed and results provided.

CM stimulus visibility was kept at approximately 7 times above detection threshold (Skerswetat, Formankiewicz and Waugh, 2016). The three visibility levels for L and LM stimuli are indicated as either ‘low’, ‘mid’, or ‘high’, which in multiples of detection threshold correspond to: L_low = 3, L_high = 98, LM_low = 3, LM_mid = 5, and LM_high = 43.

Symbols on the righthand side of each table indicate statistical significance levels (i.e. asterix * indicates p ≤ 0.05; ** indicates p ≤ 0.01; *** indicates p ≤ 0.001; **** indicates p ≤ 0.0001) and ‘n.s.’ indicates not significant (p>0.05).

- 1. **Statistical results for relative proportions (adjusted using Greenhouse-Geisser correction)**

| **Similar visible CM, L, LM** | | | | | |
| --- | --- | --- | --- | --- | --- |
| **Relative proportions [%]** | **F(df effect, df error)** | **F- value** | **p-value** | **Significance** |  |
| Stimulus type | 1.9 ,13.5 | 1.4 | 0.2794 | n.s. |  |
| Perceptual state | 1.3, 8.8 | 1.4 | 0.2800 | n.s. |  |
| Type* State | 1.9, 13.4 | 23.0 | 0.0001 | **** |  |
| **Planned comparisons** |  |  |  |  |  |
| Exclusive visibility |  |  |  |  |  |
| CMvsL | 1.0, 7.0 | 19.3 | 0.0032 | ** |  |
| CMvsLM | 1.0, 7.0 | 14.2 | 0.0070 | ** |  |
| LvsLM | 1.0, 7.0 | 6.0 | 0.0446 | * |  |
| Piecemeal |  |  |  |  |  |
| CMvsL | 1.0, 7.0 | 34.8 | 0.0006 | *** |  |
| CMvsLM | 1.0, 7.0 | 10.5 | 0.0144 | * |  |
| LvsLM | 1.0, 7.0 | 7.8 | 0.0267 | * |  |
| Superimposition |  |  |  |  |  |
| CMvsL | 1.0, 7.0 | 35.2 | 0.0006 | *** |  |
| CMvsLM | 1.0, 7.0 | 33.4 | 0.0007 | *** |  |
| LvsLM | 1.0, 7.0 | 13.7 | 0.0077 | ** |  |

*Table 1: Results for two-factor repeated measures ANOVAs with Greenhouse-Geisser corrections as well as planned comparisons for similarly visible CM, L, and LM stimulus types and the proportions of the perceptual states (i.e. in %).*

| **All L and LM** | | | | |
| --- | --- | --- | --- | --- |
| **Relative proportions [%]** | **F(df effect, df error)** | **F- value** | **p-value** | **Significance** |
| Stimulus type | 1.0, 7.0 | 1.4 | 0.2677 | n.s. |
| Visibility | 1.1, 7.4 | 0.6 | 0.4627 | n.s. |
| Perceptual state | 1.5, 10.2 | 17.0 | 0.0010 | * |
| Type * Visibility | 1.0, 7.3 | 0.8 | 0.4109 | n.s. |
| Type * State | 1.4, 10.0 | 7.7 | 0.0139 | * |
| Visibility * State | 2.3, 15.8 | 14.4 | 0.0002 | ** |
| Type * Visibility * State | 2.8, 19.4 | 6.0 | 0.0052 | ** |
| **Planned comparisons** |  |  |  |  |
| L_Low_EV vs. LM_Low_EV | 1.0, 7.0 | 4.7 | 0.0659 | * |
| L_Low_PM vs. LM_Low_PM | 1.0, 7.0 | 0.01 | 0.9115 | n.s. |
| L_Low_SI vs. LM_Low_SI | 1.0, 7.0 | 3.7 | 0.0964 | n.s. |
| L_Mid_EV vs. LM_Mid_EV | 1.0, 7.0 | 6.0 | 0.0446 | * |
| L_Mid_PM vs. LM_Mid_PM | 1.0, 7.0 | 7.8 | 0.0267 | * |
| L_Mid_SI vs. LM_Mid_SI | 1.0, 7.0 | 13.7 | 0.0077 | ** |
| L_High_EV vs. LM_High_EV | 1.0, 7.0 | 1.0 | 0.3576 | n.s. |
| L_High_PM vs. LM_High_PM | 1.0, 7.0 | 1.8 | 0.2229 | n.s. |
| L_High_SI vs. LM_High_SI | 1.0, 7.0 | 2.0 | 0.1972 | n.s. |
| L_Low_EV vs. L_Mid_EV | 1.0, 7.0 | 7.8 | 0.0266 | * |
| L_Low_PM vs. L_Mid_PM | 1.0, 7.0 | 17.3 | 0.0043 | ** |
| L_Low_SI vs. L_Mid_SI | 1.0, 7.0 | 8.6 | 0.0220 | * |
| L_Low_EV vs. L_High_EV | 1.0, 7.0 | 11.5 | 0.0115 | * |
| L_Low_PM vs. L_High_PM | 1.0, 7.0 | 27.7 | 0.0012 | ** |
| L_Low_SI vs. L_High_SI | 1.0, 7.0 | 6.0 | 0.0442 | * |
| L_Mid_EV vs. L_High_EV | 1.0, 7.0 | 9.5 | 0.0177 | * |
| L_Mid_PM vs. L_High_PM | 1.0, 7.0 | 19.6 | 0.0031 | ** |
| L_Mid_SI vs. L_High_SI | 1.0, 7.0 | 2.0 | 0.2020 | n.s. |
| LM_Low_EV vs. LM_Mid_EV | 1.0, 7.0 | 1.3 | 0.2981 | n.s. |
| LM_Low_PM vs. LM_Mid_PM | 1.0, 7.0 | 0.4 | 0.5451 | n.s. |
| LM_Low_SI vs. LM_Mid_SI | 1.0, 7.0 | 0.8 | 0.4106 | n.s. |
| LM_Low_EV vs. LM_High_EV | 1.0, 7.0 | 6.0 | 0.0443 | * |
| LM_Low_PM vs. LM_High_PM | 1.0, 7.0 | 18.0 | 0.0038 | ** |
| LM_Low_SI vs. LM_High_SI | 1.0, 7.0 | 7.6 | 0.0284 | * |
| LM_Mid_EV vs. LM_High_EV | 1.0, 7.0 | 1.7 | 0.2360 | n.s. |
| LM_Mid_PM vs. LM_High_PM | 1.0, 7.0 | 15.3 | 0.0058 | ** |
| LM_Mid_SI vs. LM_High_SI | 1.0, 7.0 | 15.2 | 0.0059 | ** |

*Table 2: Results for three-factor repeated measures ANOVAs with Greenhouse-Geisser corrections as well as planned comparisons for L and LM stimulus types, three different visibility levels, and the proportions of the perceptual states (i.e in %).*

- 1. **Statistical results for mean durations**

| **Similar visible CM, L, LM** | | | | |
| --- | --- | --- | --- | --- |
| **Mean durations [sec]** | **F(df effect, df error)** | **F- value** | **p-value** | **Significance** |
| Stimulus type | 1.1, 7.8 | 8.5 | 0.0180 | * |
| Perceptual state | 1.1, 7.4 | 6.2 | 0.0389 | * |
| Stimulus type* Perceptual state | 1.1, 7.9 | 9.3 | 0.0143 | * |
| **Planned comparisons** | | | | |
| Exclusive visibility |  |  |  |  |
| CMvsL | 1.0, 7.0 | 17.1 | 0.0044 | ** |
| CMvsLM | 1.0, 7.0 | 8.8 | 0.0209 | * |
| LvsLM | 1.0, 7.0 | 2.9 | 0.1332 | n.s. |
| Piecemeal |  |  |  |  |
| CMvsL | 1.0, 7.0 | 0.2 | 0.6472 | n.s. |
| CMvsLM | 1.0, 7.0 | 0.4 | 0.5381 | n.s. |
| LvsLM | 1.0, 7.0 | 6.4 | 0.0387 | * |
| Superimposition |  |  |  |  |
| CMvsL | 1.0, 7.0 | 10.6 | 0.0138 | * |
| CMvsLM | 1.0, 7.0 | 8.0 | 0.0253 | * |
| LvsLM | 1.0, 7.0 | 5.5 | 0.0521 | n.s. |

*Table 3: Results for two-factor repeated measures ANOVAs with Greenhouse-Geisser corrections as well as planned comparisons for similarly visible CM, L, and LM stimulus types and their mean durations of the perceptual states in seconds.*

| **All L and LM** | | | | |
| --- | --- | --- | --- | --- |
| **Mean durations [sec]** | **F(df effect, df error)** | **F- value** | **p-value** | **Significance** |
| Stimulus type | 1.0, 7.0 | 2.8 | 0.1359 | n.s. |
| Visibility | 1.3, 8.8 | 1.3 | 0.3039 | n.s. |
| Perceptual state | 1.2, 8.5 | 12.4 | 0.0055 | ** |
| Type*Visibility | 1.3, 9.0 | 4.5 | 0.0566 | n.s. |
| Type*State | 1.1, 7.9 | 6.6 | 0.0310 | * |
| Visibility * State | 1.9, 13.5 | 10.6 | 0.0018 | ** |
| Type * Visibility * State | 2.6, 18.5 | 3.0 | 0.0622 | n.s. |
| **Planned comparisons** | | | | |
| L_Low_EV vs. LM_Low_EV | 1.0, 7.0 | 0.4 | 0.5702 | n.s. |
| L_Low_PM vs. LM_Low_PM | 1.0, 7.0 | 4.3 | 0.0779 | n.s. |
| L_Low_SI vs. LM_Low_SI | 1.0, 7.0 | 4.6 | 0.0687 | n.s. |
| L_Mid_EV vs. LM_Mid_EV | 1.0, 7.0 | 2.9 | 0.1332 | n.s. |
| L_Mid_PM vs. LM_Mid_PM | 1.0, 7.0 | 6.4 | 0.0387 | * |
| L_Mid_SI vs. LM_Mid_SI | 1.0, 7.0 | 5.5 | 0.0521 | n.s. |
| L_High_EV vs. LM_High_EV | 1.0, 7.0 | 2.9 | 0.1344 | n.s. |
| L_High_PM vs. LM_High_PM | 1.0, 7.0 | 1.5 | 0.2613 | n.s. |
| L_High_SI vs. LM_High_SI | 1.0, 7.0 | 1.3 | 0.2924 | n.s. |
| L_Low_EV vs. L_Mid_EV | 1.0, 7.0 | 2.1 | 0.1930 | n.s. |
| L_Low_PM vs. L_Mid_PM | 1.0, 7.0 | 6.8 | 0.0349 | * |
| L_Low_SI vs. L_Mid_SI | 1.0, 7.0 | 13.1 | 0.0084 | ** |
| L_Low_EV vs. L_High_EV | 1.0, 7.0 | 5.7 | 0.0487 | * |
| L_Low_PM vs. L_High_PM | 1.0, 7.0 | 9.4 | 0.0182 | * |
| L_Low_SI vs. L_High_SI | 1.0, 7.0 | 38.8 | 0.0004 | *** |
| L_Mid_EV vs. L_High_EV | 1.0, 7.0 | 8.8 | 0.0211 | * |
| L_Mid_PM vs. L_High_PM | 1.0, 7.0 | 5.8 | 0.0469 | * |
| L_Mid_SI vs. L_High_SI | 1.0, 7.0 | 0.7 | 0.4268 | n.s. |
| LM_Low_EV vs. LM_Mid_EV | 1.0, 7.0 | 1.5 | 0.2582 | n.s. |
| LM_Low_PM vs. LM_Mid_PM | 1.0, 7.0 | 1.1 | 0.3236 | n.s. |
| LM_Low_SI vs. LM_Mid_SI | 1.0, 7.0 | 0.5 | 0.4963 | n.s. |
| LM_Low_EV vs. LM_High_EV | 1.0, 7.0 | 1.9 | 0.2076 | n.s. |
| LM_Low_PM vs. LM_High_PM | 1.0, 7.0 | 5.3 | 0.0552 | n.s. |
| LM_Low_SI vs. LM_High_SI | 1.0, 7.0 | 9.7 | 0.0172 | * |
| LM_Mid_EV vs. LM_High_EV | 1.0, 7.0 | 1.6 | 0.2452 | n.s. |
| LM_Mid_PM vs. LM_High_PM | 1.0, 7.0 | 1.6 | 0.2452 | n.s. |
| LM_Mid_SI vs. LM_High_SI | 1.0, 7.0 | 14.9 | 0.0063 | ** |

*Table 4: Results for three-factor repeated measures ANOVAs with Greenhouse-Geisser corrections as well as planned comparisons for L and LM stimulus types, three different visibility levels, and the mean durations of the perceptual states in seconds.*

- 1. **Statistical results for perceptual alternations**

| **Similar visible CM, L, LM** | | | | |
| --- | --- | --- | --- | --- |
| **Perceptual alternations**  **[flips per trial duration]** | **F(df effect, df error)** | **F- value** | **p-value** | **Significance** |
| Type | 1.9, 13.2 | 50.8 | 0.0000 | **** |
| Flip | 1.0, 7.0 | 50.0 | 0.0002 | *** |
| Type*Flip | 1.5, 10.6 | 74.9 | 0.0000 | **** |
| **Planned Comparisons** | | | | |
| CM_fullflips vs L_fullflips | 1.0, 7.0 | 5.1 | 0.0589 | n.s. |
| CM_halfflips vs L_halflfips | 1.0, 7.0 | 203.9 | 0.0000 | **** |
| CM_fullflips vs LM_fullflips | 1.0, 7.0 | 3.2 | 0.1188 | n.s. |
| CM_halfflips vs LM_halflfips | 1.0, 7.0 | 65.7 | 0.0001 | **** |
| L_fullflips vs LM_fullflips | 1.0, 7.0 | 4.5 | 0.0704 | n.s. |
| L_halfflips vs LM_halflfips | 1.0, 7.0 | 3.0 | 0.1277 | n.s. |
| CM_fullflips vs CM_Halfflips | 1.0, 7.0 | 12.0 | 0.0105 | * |
| L_fullflips vs L_Halfflips | 1.0, 7.0 | 90.4 | 0.0000 | **** |
| LM_fullflips vs LM_Halfflips | 1.0, 7.0 | 53.5 | 0.0002 | *** |

*Table 5: Results for two-factor repeated measures ANOVAs with Greenhouse-Geisser corrections as well as planned comparisons for similarly visible CM, L, and LM stimulus types, and the perceptual change types, i.e. full flips and half flips, in flips per trial duration.*

| **All L and LM** | | | | |
| --- | --- | --- | --- | --- |
| **Perceptual alternations**  **[flips per trial duration]** | **F(df effect, df error)** | **F- value** | **p-value** | **Significance** |
| Type | 1.0, 7.0 | 12.8 | 0.0091 | ** |
| Visibility | 1.3, 9.1 | 2.3 | 0.1628 | n.s. |
| Flip | 1.0, 7.0 | 72.0 | 0.0001 | **** |
| Type * Visibility | 1.7, 11.7 | 2.9 | 0.1027 | n.s. |
| Type * Flip | 1.0, 7.0 | 1.0 | 0.3539 | n.s. |
| Visibility * Flip | 1.4, 9.5 | 1.9 | 0.2021 | n.s. |
| Type * Visibility * Flip | 1.3, 9.2 | 0.6 | 0.5038 | n.s. |

*Table 6: Results for three-factor repeated measures ANOVAs with Greenhouse-Geisser corrections as well as planned comparisons for L and LM stimulus types, three different visibility levels, and the perceptual change type, i.e. full flip (ff) and half flip (hf), in flips per trial duration.*

- 1. **Statistical results for the CM control experiment**

| **Relative proportions [%]** | | | | | |
| --- | --- | --- | --- | --- | --- |
| Repeated measures. GLM, GG(two factors) | **F(df effect,df error)** | | **F- value** | **p** | **Significance** |
| Visibility | 1.0, 3.0 | 1 | | 0.3910 | n.s. |
| Perceptual state | 1.0, 3.0 | 9.8 | | 0.0516 | * |
| Visibility*State | 1.0, 3.0 | 3.3 | | 0.1685 | n.s. |
| **Mean duration [sec]** | | | | | |
| Visibility | 1.0, 3.0 | 0.001 | | 0.9763 | n.s. |
| Perceptual state | 1.6,4.9 | 1.7 | | 0.2709 | n.s. |
| Visibility*State | 1.4,4.1 | 2.5 | | 0.1928 | n.s. |
| **Perceptual changes [flips per trial duration]** | | | | | |
| Visibility | 1.0, 3.0 | 1.1 | | 0.3723 | n.s. |
| Flip type | 1.0, 3.0 | 2.3 | | 0.2297 | n.s. |
| Visibility *Flip | 1.0, 3.0 | 1.1 | | 0.3724 | n.s. |

*Table 7: Results for two-factor repeated measures ANOVAs with Greenhouse-Geisser corrections of the two visibility levels for CM stimuli (3.5x vs. 3.5x and 7x vs. 7x), and the relative results (i.e. %), mean duration (i.e. sec), and perceptual alternations (i.e. flips per trial duration), here either full or separately half flips in flips per trial durations.*

1. **Second experiment: Statistical results for Levelt’s four laws using an IOG paradigm**

Below are statistical outcomes for the CM vs. LM as well as the (control) LM vs. LM experimental conditions used to test whether Levelt’s laws apply to the IOG paradigm. Visibility of the CM stimulus was kept at 7 times above detection threshold (Skerswetat, Formankiewicz and Waugh, 2016). Visibilities used to test the first, second and third law were approximately 43, 7, and 3.5 times above threshold, indicated as ‘High’, ‘Mid’, and ‘Low’, respectively. To test the fourth law, CM vs. LM visibility levels were approximately 3.5 vs. 3.5 and 7 vs. 7 times above threshold. The same visibilities as well as approximately 43 vs. 43 times threshold were used for the LM vs LM condition.

- 1. **Statistical results for Levelt’s first law**

| **CMvsLM** | | | | |
| --- | --- | --- | --- | --- |
| **Relative proportions [%]** | **F(df effect,df error)** | **F- value** | **p** | **Significance** |
| Type | 1.0, 8.0 | 27.4 | 0.0008 | *** |
| Visibility | 1.3541812, 10.8334496 | 38.4 | 0.0000 | **** |
| Type*Visibility | 1.2, 9.3 | 17.3 | 0.0018 | *** |
| **LMvsLM** | |  |  |  |
| Eye | 1.0, 8.0 | 0.3 | 0.6155 | n.s. |
| Visibility | 1.5 11.6 | 8.7 | 0.0079 | *** |
| Eye* Visibility | 1.1, 8.6 | 24.8 | 0.0008 | *** |
| **Planned comparisons** | | | | |
| **[fixed vs. variable]** |  |  |  |  |
| CM_mid vs. LM_low | 1.0, 8.0 | 26.4 | 0.0009 | *** |
| CM_mid vs. LM_mid | 1.0, 8.0 | 22.0 | 0.0016 | ** |
| CM_mid vs. LM_high | 1.0, 8.0 | 2.1 | 0.1811 | n.s. |
| LM_mid vs. LM_low | 1.0, 8.0 | 37.4 | 0.0003 | *** |
| LM_mid vs. LM_mid | 1.0, 8.0 | 0.9 | 0.3751 | n.s. |
| LM_mid vs. LM_high | 1.0, 8.0 | 17.1 | 0.0033 | ** |

*Table 8: Results for two-factor repeated measures ANOVAs with Greenhouse-Geisser corrections as well as planned comparisons of stimulus types for CM vs. LM and LM vs. LM condition, and the proportions of the exclusive visibility in %. As the LM vs LM uses the same stimulus type, we refer here to the ‘Eye’ (i.e. fixed vs. variable) rather than the stimulus type.*

- 1. **Statistical results for Levelt’s second law**

| **CMvsLM (all subjects)** | | | | | | |
| --- | --- | --- | --- | --- | --- | --- |
| **Mean durations [sec]** | | **F(df effect,df error)** | | **F- value** | **p** | **Significance** |
| Type | | 1.0, 8.0 | | 4.1 | 0.0777 | n.s. |
| Visibility | | 1.0, 8.0 | | 5.1 | 0.0538 | * |
| Type * Visibility | | 1.0, 8.2 | | 4.3 | 0.0699 | n.s. |
| **LMvsLM (all subjects)** | | | | | | |
| Eye | | 1.0, 8.0 | | 0.3 | 0.5829 | n.s. |
| Visibility | | 1.0, 8.3 | | 1.3 | 0.2943 | n.s. |
| Eye* Visibility | | 1.0, 8.0 | | 1.5 | 0.2486 | n.s. |
| **CMvsLM (Without outliers)** | | | |  |  | |
| Type | 1.0, 6.0 | | | 43.3 | 0.0006 | *** |
| Visibility | 1.0, 6.3 | | | 20.6 | 0.0034 | ** |
| Type * Visibility | 1.1, 6.8 | | | 25.3 | 0.0014 | ** |
| **LMvsLM (Without outliers)** | | | | | | |
| Type | 1.0, 6.0 | | | 1.6 | 0.2486 | n.s. |
| Visibility | 1.0, 6.0 | | | 12.5 | 0.0030 | ** |
| Type * Visibility | 1.0, 6.0 | | | 31.5 | 0.0004 | *** |
| Planned comparison | | | | | | |
| CM_mid vs. LM_low | | | 1.0, 6.0 | 34.2 | 0.0011 | ** |
| CM_mid vs. LM_mid | | | 1.0, 6.0 | 16.6 | 0.0065 | ** |
| CM_mid vs. LM_high | | | 1.0, 6.0 | 0.6 | 0.4641 | n.s. |
| LM_mid vs. LM_low | | | 1.0, 6.0 | 38.8 | 0.0008 | *** |
| LM_mid vs. LM_mid | | | 1.6, 9.5 | 8.6 | 0.0259 | * |
| LM_mid vs. LM_high | | | 1.3, 7.5 | 19.9 | 0.0043 | ** |

*Table 9: Results for two-factor repeated measures ANOVAs with Greenhouse-Geisser corrections of stimulus types for CM vs. LM and LM vs. LM conditions, and the mean durations of exclusive visibility (i.e. in seconds). As the LM vs LM uses the same stimulus type, we refer here to the ‘Eye’ (i.e. fixed vs. variable) rather than the stimulus type.*

- 1. **Statistical results for Levelt’s third law**

| **Full-flips** | | | | | | |
| --- | --- | --- | --- | --- | --- | --- |
| **CMvsLM** | **F(df effect, df error)** | | | **F- value** | **p** | **Significance** |
| Visibility | | 1.3, 10.8 | | 2.5 | 0.1408 | n.s. |
| **LMvsLM** | |  | |  |  |  |
| Visibility | | 1.4, 11.4 | | 1.6 | 0.2393 | n.s. |
| **Half-flips** | | | | | | |
| **CMvsLM** | |  | |  |  |  |
| Visibility | | 1.8, 14.3 | | 5.1 | 0.0239 | * |
| **Planned comparisons** | |  | |  |  |  |
| CM_midLM_low vs CM_midLM_mid | | 1.0, 8.0 | 2.9 | | 0.1290 | n.s. |
| CM_midLM_low vs CM_midLM_high | | 1.0, 8.0 | 8.8 | | 0.0179 | * |
| CM_midLM_mid vs CM_midLM_high | | 1.0, 8.0 | 2.8 | | 0.1347 | n.s. |
| **LMvsLM** | |  |  | |  |  |
| Visibility | | 1.1, 9.1 | 0.9 | | 0.3923 | n.s. |

*Table 10: Results for one-factor repeated measures ANOVAs with Greenhouse-Geisser corrections as well as planned comparisons of stimulus types for CM vs. LM and LM vs. LM condition and different visibility levels. The perceptual alternations are either full flips or half flips (i.e. in flips per trial durations).*


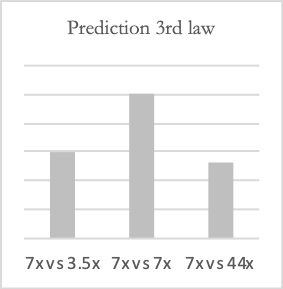

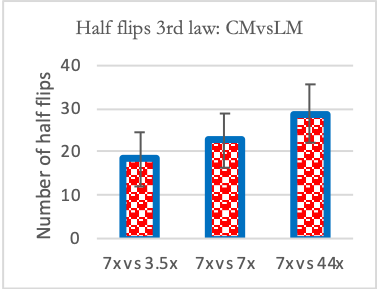

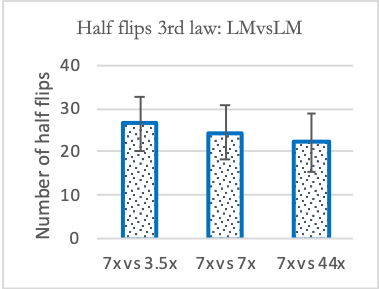


*Figure 1: Results for Levelt’s third law when using half-flips (all alternations that are not full-flips)*.


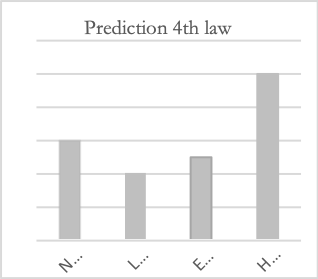

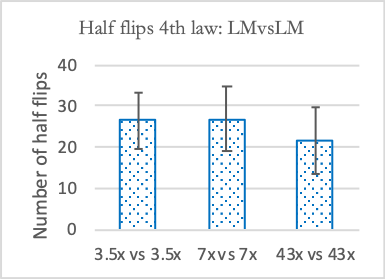

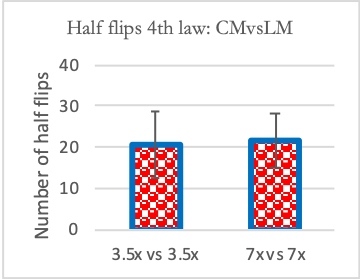


*Figure 2: Results for Levelt’s fourth law when using half-flips (all alternations that are not full-flips)*.

- 1. **Statistical results for Levelt’s fourth law**

| **Full-flips** | | | | | |
| --- | --- | --- | --- | --- | --- |
| **CMvsLM** | **F(df effect, df error)** | | **F- value** | **p** | **Significance** |
| Visibility | | 1.0, 8.0 | 1.3 | 0.2901 | n.s. |
| **LMvsLM** | |  |  |  |  |
| Visibility | | 1.4, 10.8 | 0.1 | 0.8163 | n.s. |
| **Half-flips** | | | | | |
| **CMvsLM** | |  |  |  |  |
| Visibility | | 1.0, 8.0 | 0.1 | 0.7723 | n.s. |
| **LMvsLM** | |  |  |  |  |
| Visibility | | 1.4, 11.2 | 1.0 | 0.3786 | n.s. |

*Table 11: Results for one-factor repeated measures ANOVAs with Greenhouse-Geisser corrections of stimulus types for CM vs. LM and LM vs. LM condition, and perceptual alternations, here either full- or separately half-flips in flips per trial durations.*

## **3.0 Analysis across time**

Session 1) First vs. fourth trial (Averages across participants with standard errors)

| **Type** | **IOG Exclusivity [%]** | **Piecemeal [%]** | **Superimposition [%]** |
| --- | --- | --- | --- |
| L98x: | 32.8 ± 5.5 vs. 35.6 ± 5.2 | 61.9 ± 6.9 vs. 59.1 ± 4.4 | 5.2 ± 3.0 vs. 5.3 ± 3.7 |
| CM7x: | 8.5 ± 3.6 vs. 2.5 ± 1.1 | 26.6 ± 11.2 vs. 35.6 ± 11.6 | 64.9 ± 13.9 vs. 61.9 ± 12.6 |

Session 2) First vs. fourth trial

| **Type** | **IOG Exclusivity [%]** | **Piecemeal [%]** | **Superimposition [%]** |
| --- | --- | --- | --- |
| L98x: | 33.6 ± 8.5 vs. 41.1 ± 5.7 | 48.8 ± 9.2 vs. 56.2 ±4.7 | 5.1 ± 4.5 vs. 2.8 ± 1.6 |
| CM7x: | 7.9 ± 5.5 vs. 10.5 ± 9.4 | 20.0 ± 9.8 vs. 18.0 ± 8.1 | 59.6 ± 16.2 vs. 71.5 ± 13.0 |

*Table 12: Relative proportions across trials and sessions. A repeated measures ANOVA across stimulus type, state, trials, and sessions found no significant effect of trials [F(1,7)=2.0,p=0.20] or sessions [F(1,7)=0.2,p=0.68].*

|  | | | | | |
| --- | --- | --- | --- | --- | --- |
| **L98vsCM** | **F(df effect, df error)** | | **F- value** | **p** | **Significance** |
| Stimulus type | | 1.0, 7.0 | 1.0 | 0.3506 | n.s. |
| Sessions | | 1.0, 7.0 | 0.2 | 0.6845 | n.s. |
| Perceptual states | | 1.2,8.6 | 10.2 | 0.0091 | ** |
| Trials | | 1.0, 7.0 | 2.0 | 0.1970 | n.s. |
| Types*Sessions | | 1.0, 7.0 | 1.0 | 0.3506 | n.s. |
| Types*States | | 1.0, 7.0 | 36.8 | 0.0003 | *** |
| Sessions *States | | 1.3,9.2 | 3.0 | 0.1124 | n.s. |
| Types*Trials | | 1.0, 7.0 | 1.0 | 0.3506 | n.s. |
| Sessions*Trials | | 1.0, 7.0 | 0.2 | 0.6845 | n.s. |
| States*Trials | | 3.0,21.1 | 0.8 | 0.5200 | n.s. |
| Types*Sessions*States | | 1.7,11.7 | 1.3 | 0.3015 | n.s. |
| Types*Sessions*Trials | | 1.0, 7.0 | 1.0 | 0.3506 | n.s. |
| Types*States*Trials | | 2.3,15.9 | 2.3 | 0.1324 | n.s. |
| Sessions*States*Trialss | | 2.7,19.0 | 1.0 | 0.3896 | n.s. |
| Types*Sessions*States*Trial | | 2.4,17.0 | 0.7 | 0.5421 | n.s. |
|  | |  |  |  |  |

*Table 13: Results for multi-factor repeated measures ANOVAs with Greenhouse-Geisser corrections for L98 vs. CM. for an analysis across stimulus types, perceptual states, trials, and sessions.*

**References**

Skerswetat, J., Formankiewicz, M. A. and Waugh, S. J. (2016) ‘Very few exclusive percepts for contrast-modulated stimuli during binocular rivalry’, Vision Research. Elsevier Ltd, 121, pp. 10–22. doi: 10.1016/j.visres.2016.01.002.
